# Detection of *Salmonella* Typhi bacteriophages in surface waters as a scalable approach to environmental surveillance

**DOI:** 10.1371/journal.pntd.0011912

**Published:** 2024-02-08

**Authors:** Sneha Shrestha, Kesia Esther Da Silva, Jivan Shakya, Alexander T. Yu, Nishan Katuwal, Rajeev Shrestha, Mudita Shakya, Sabin Bikram Shahi, Shiva Ram Naga, Christopher LeBoa, Kristen Aiemjoy, Isaac I. Bogoch, Senjuti Saha, Dipesh Tamrakar, Jason R. Andrews

**Affiliations:** 1 Center for Infectious Disease Research and Surveillance, Dhulikhel Hospital Kathmandu University Hospital, Kavre, Nepal; 2 Research and Development Division, Dhulikhel Hospital Kathmandu University Hospital, Kavre, Nepal; 3 Stanford University, Department of Medicine, Division of Infectious Diseases and Geographic Medicine, Stanford, California, United States of America; 4 Institute for Research in Science and Technology, Kathmandu, Nepal; 5 Department of Pharmacology, Kathmandu University School of Medical Sciences, Kathmandu, Nepal; 6 University of California Berkeley, Department of Environmental Health Sciences, Berkeley, California, United States of America; 7 University of California Davis, School of Medicine, Department of Public Health Sciences, Davis, California, United States of America; 8 Department of Medicine, Division of Infectious Diseases, University of Toronto, Toronto, Canada; 9 Child Health Research Foundation, Dhaka, Bangladesh; 10 Department of Community Medicine, Kathmandu University School of Medical Sciences, Kathmandu, Nepal; University of Connecticut, UNITED STATES

## Abstract

**Background:**

Environmental surveillance, using detection of *Salmonella* Typhi DNA, has emerged as a potentially useful tool to identify typhoid-endemic settings; however, it is relatively costly and requires molecular diagnostic capacity. We sought to determine whether *S*. Typhi bacteriophages are abundant in water sources in a typhoid-endemic setting, using low-cost assays.

**Methodology:**

We collected drinking and surface water samples from urban, peri-urban and rural areas in 4 regions of Nepal. We performed a double agar overlay with *S*. Typhi to assess the presence of bacteriophages. We isolated and tested phages against multiple strains to assess their host range. We performed whole genome sequencing of isolated phages, and generated phylogenies using conserved genes.

**Findings:**

*S*. Typhi-specific bacteriophages were detected in 54.9% (198/361) of river and 6.3% (1/16) drinking water samples from the Kathmandu Valley and Kavrepalanchok. Water samples collected within or downstream of population-dense areas were more likely to be positive (72.6%, 193/266) than those collected upstream from population centers (5.3%, 5/95) (p=0.005). In urban Biratnagar and rural Dolakha, where typhoid incidence is low, only 6.7% (1/15, Biratnagar) and 0% (0/16, Dolakha) river water samples contained phages. All *S*. Typhi phages were unable to infect other *Salmonella* and non-*Salmonella* strains, nor a Vi-knockout *S*. Typhi strain. Representative strains from *S*. Typhi lineages were variably susceptible to the isolated phages. Phylogenetic analysis showed that *S*. Typhi phages belonged to the class *Caudoviricetes* and clustered in three distinct groups.

**Conclusions:**

*S*. Typhi bacteriophages were highly abundant in surface waters of typhoid-endemic communities but rarely detected in low typhoid burden communities. Bacteriophages recovered were specific for *S*. Typhi and required Vi polysaccharide for infection. Screening small volumes of water with simple, low-cost (~$2) plaque assays enables detection of *S*. Typhi phages and should be further evaluated as a scalable tool for typhoid environmental surveillance.

## Author summary

Typhoid fever is a major health issue in low- and middle-income countries, causing illness and death. The World Health Organization recommends the use of typhoid-conjugate vaccines to combat its spread. However, limited data on typhoid prevalence in these countries hinders effective vaccination programs. Environmental surveillance, which involves detecting *Salmonella* Typhi DNA in the environment, has emerged as a method to identify high-risk areas for typhoid transmission. Nevertheless, this approach is costly and requires infrastructure often unavailable in many communities. To address this, we conducted a study in Nepal to explore a low-cost and sustainable typhoid surveillance method. We examined typhoid bacteriophages in surface and drinking water across communities with varying population densities and typhoid burdens. Phages were easily detectable in communities with high typhoid burdens but less prevalent in areas with lower burdens. The isolated phages exhibited specificity for *S*. Typhi and were effective against different bacterial strains in Nepal. This study demonstrates that phage-based assays can be a simple and affordable tool for typhoid environmental surveillance. This method can help identify areas that require vaccination prioritization and interventions for clean water and sanitation. By utilizing phage-based assays, health authorities can better understand typhoid transmission and implement targeted control measures.

## 1. Introduction

Typhoid fever is a bacterial infection caused by the Gram-negative bacterium *Salmonella enterica* subspecies enterica serovar Typhi (*S*. Typhi) and is usually contracted by ingestion of contaminated food or water [1]. An estimated 11 million cases of typhoid fever occur annually worldwide causing more than 100,000 deaths each year [2] The highest burden occurs in low- and middle-income countries (LMICs) with poor access to improved water supply and sanitation [3]. Successful management of typhoid fever using antibiotics is becoming increasingly difficult due to drug resistance [4]. The continued emergence and rapid expansion of antimicrobial resistance to *S*. Typhi have spurred accelerated efforts to roll out new conjugate vaccines for the prevention of typhoid infection in high-risk populations [5].

Routine typhoid fever vaccination use has been very limited in the past, even in endemic areas. Recently the World Health Organization (WHO) recommended the introduction of typhoid conjugate vaccines (TCV) in countries with the highest burden of typhoid disease or a high burden of antimicrobial-resistant *S*. Typhi [6]. However, the scarcity of data on typhoid burden in many at-risk countries poses a major hurdle to the adoption of TCV in national immunization programs [7]. Currently, the estimates of typhoid fever burden have been generated via short-term population-based research studies, and these data are frequently geographically and temporally limited [8]. Given the current lack of data and the challenges faced in population-based surveillance, environmental surveillance is emerging as an important tool in the fight against typhoid fever [9].

Previous studies have shown environmental surveillance as a tool to identify high-risk settings for enteric fever transmission [8–10]. Despite the clear role of contaminated water in the transmission of typhoid, detection of the organisms has proven challenging, with *S*. Typhi difficult to isolate by culture methods from water and other environmental samples (8). Molecular detection of *S*. Typhi DNA is now the primary method for detecting *S*. Typhi in the environment, but requires molecular laboratory infrastructure and trained staff, and estimated costs are hundreds of dollars per sample [11].

Bacteriophages (hereinafter referred to as phages) are viruses that infect bacteria. Their abundance and host specificity make them a promising alternative or complimentary tool for the environmental monitoring of pathogenic bacteria [12]. S. Typhi phages have been characterized for decades, comprised the major typing system for the bacteria (based on their susceptibility to phage panels), and were used clinically as treatment prior to the discovery of chloramphenicol. One study from 1930 found that S. Typhi phages were abundant and seasonal in surface waters in Kolkata [13]. However, few studies have characterized the distribution and abundance of *S*. Typhi phages in typhoid-endemic settings or examined their genetic composition and diversity.

In this study, we investigated the presence and abundance of typhoid phages in surface and drinking water across diverse communities in Nepal with differing population density and typhoid burden. In addition to phage detection, we performed genome sequencing to provide a comprehensive understanding of the genetic diversity of S. Typhi phages. By incorporating genome sequencing into our investigation, we aim to bridge this knowledge gap, allowing us to elucidate distribution patterns across these diverse communities. We hypothesized that *S*. Typhi phages would be readily detectable, using simple, low-cost assays, in the surface waters of communities with high typhoid burden and not frequently detected in communities with lower typhoid burden.

## 2. Methods

### Bacterial strains, Vi phages and culture conditions

A complete list of strains used in this study can be found in S1 Table. The attenuated *Salmonella enterica* serovar Typhi strain BRD948 was used for propagation. This strain is not only heavily capsulated but is also attenuated and can be used in a containment level 2 environment [14]. Bacteria were cultured using Tryptic Soy Broth (TSB) (HiMedia Laboratories, USA), at 37°C with aeration, supplemented with agar as required. The optical density of liquid culture was adjusted to a 0.5 McFarland standard. Phages used in this paper as a control for the experiments were isolated from clinical samples and correspond to a *S*. Typhi Vi phage collection from the class *Caudoviricetes*, designated types II, III, IV, V, VI, VII. Vi phages were obtained from Cambridge University, Cambridge, United Kingdom, but the original sources of these phages date from the 1930s through 1955 [15].

### Sampling sites

Between November 2019 and May 2022, environmental water samples were collected from the Kathmandu Valley and three other districts of Nepal (Kavrepalanchok, Dolakha and Morang). We collected river water samples from 5 major rivers and a combined river segment in Kathmandu Valley and Kavrepalanchok monthly from November 2019 to July 2021 (except in April and May 2020 due to government-imposed lockdown during the COVID-19 pandemic). A total of 19 different sites, 16 in Kathmandu valley and 3 in Kavrepalanchok, each separated by approximately 5 km, located upstream and downstream from the river confluence were sampled. This sampling strategy let us sample the upstream sites with relatively sparse settlement, the densely populated central settlement area and also downstream sites mostly located around agricultural areas. Sampling was monthly repeated every 30 usually between 8am and 12pm.

Within Kathmandu valley, the majority of the sites (10/16) were either located in rivers inside, around or downstream to Kathmandu Metropolitan city, the capital city of Nepal, with a population of 1,571,000 and population density of 17,103 people per km^2^. Unmanaged sewage, waste disposal and lack of their proper regulation in this highly dense area has led to their direct disposal into the river system in Kathmandu. Wastewater management is under the responsibility of Kathmandu Upatyaka Khanepani Limited. Until recently, most of the sewage in the Bagmati River and its tributaries was directly released without treatment. The Guheshwori Wastewater Treatment Plant, which began operation in October 2020, treats wastewater by chlorination for disinfection, followed by dichlorination before release back into the river.

We also sampled a river flowing through Banepa, a peri-urban city in Kavrepalanchok district with population of 67,690 and population density of 1,231 people per km^2^, just outside the Kathmandu Valley, which similarly has untreated waste and sewage disposal into the river system. Drinking water samples were collected from both Kathmandu and Kavre, from tap (direct supply from municipal storage or stored in tanks, bought from vendors outside Kathmandu) or ground-well of 16 different households. In Kathmandu drinking water supplied through the tap water system is treated with a chlorination process in treatment plants. This chlorination method is a common disinfection technique used to ensure the safety of the drinking water supply. However, it’s important to note that alternative sources of drinking water, such as water from jars, bottles, and tankers, are frequently used in Kathmandu due to water scarcity. These alternative sources may pose challenges related to contamination and may lack the same level of treatment and quality assurance as the municipal water supply. In Kavre, the Dhulikhel water treatment plant utilizes a chlorination process for disinfection. This treated water is supplied to approximately 3,000 households in the region. Given the high demand for water in Kavre, commercial jars and tankers also play a significant role in meeting the water needs of the population. Previous studies have demonstrated a very high incidence of typhoid in both communities [7].

In Dolakha, sampling was performed in the month of October 2021, in sparsely populated, mostly rural village areas with population of 172,767 and the population density is estimated to be 262 people per km^2^ and there was minimal or no visible sewage contamination. All samples collected were from river water around settlement areas, with only a few sites selected being farther from human settlement. A previous study in Dolakha found typhoid to be an uncommon cause of acute febrile illness [16].

Biratnagar, in Morang district in Southeastern Nepal, is another metropolitan city in Nepal with a population of 243,927 and population density of 3,168 people per km^2^. Samples were collected from river sites upstream, around, and downstream of the settlement area, from man-made river canals which were originally created for irrigation purposes and from tap or tube wells of households that used it for drinking along with other purposes. Most of the tube well samples were collected from poor slum communities. In Biratnagar, only one wastewater treatment plant serves a limited number of households, leaving a large portion of the city without access to wastewater treatment. No studies of typhoid incidence or prevalence among febrile patients have been reported from Biratnagar. In our review of blood culture results from three largest hospitals (one government and two private hospitals) in the city, *S*. Typhi was very rarely recovered, and clinicians reported that typhoid cases were relatively uncommon.

### Sample collection and filtrate preparation

We collected 50 mL samples of water from the surface, against river current, in a sterile bottle and placed them into separate bags on ice and transported them to the laboratory for further processing. A field negative control was collected by pouring sterile distilled water into a bottle. We performed a turbidity test using a densitometer (DEN-1, Grant Instruments Ltd., England), and samples were passed through a 0.22 μm syringe filter (Axiva, India) to obtain the filtrate that was used for evaluating the presence of phages against *S*. Typhi. The filtered sample was stored at 4°C until further use. At each sampling site, team members collected information on abiotic factors that may affect bacterial or viral survival, including presence of sewage pipes, open drain water carrying liquid and solid waste, and potential fecal exposure. Water pH, temperature, and oxygen reduction potential were also evaluated using Apera Instruments AI311. Team members also recorded whether humans or animals were interacting with the water and the type of interaction that was observed (S2 Table). All information was recorded in REDCap (v5.19.15).

### Bacteriophage isolation and amplification

Phage screening was performed by a double agar overlay method [17]. For each sample, 1 ml of filtrate was added to 100 μl of overnight liquid bacterial culture and incubated at room temperature for 10 min to allow phage absorption. A positive control was prepared with 100 μl of Typhi-specific phage stock and 100 μl of overnight liquid bacterial culture. Negative control tube was prepared with 1 ml of field negative control filtrate and 100 μl of overnight liquid bacterial culture. Molten Tryptic Soy Agar (0.7% agar) (3 mL) was added to the filtrate-bacteria mix and poured over solid hard nutrient agar base. Plates were incubated at 37°C overnight and the presence of phages resulted in the production of visible plaques (zones of lysis) in a confluent lawn of the host bacterium. Individual plaques were selected from screening plates based on their morphology and size. A total of 67 phages were isolated from all the positive screening plates.

Plaques were picked using sterile tips and resuspended in 200 μl SM buffer. Soft agar overlay lawn culture of *S*. Typhi was prepared and 10 μl of plaque-SM buffer solution was streaked across the *S*. Typhi lawn culture to obtain isolated plaques. Only isolated plaques were processed further. To obtain high concentration stocks of isolated phages, we performed propagation assays using the double agar overlay method described above. Following overnight incubation clear zones were scraped and immersed in a 5ml SM buffer and kept for one hour at 4°C. The solution was then centrifuged at 4,000 rpm for 20 min and the supernatant was passed through a 0.22 μm syringe filter to obtain a sterile phage stock solution. Finally, the titer of stock solution was obtained by plating serial dilutions of the stock in a confluent lawn of the host bacterium and counting visible plaques to determine the approximate ratio of plaque-forming units per milliliter (PFU mL^-1^).

### Determination of phage host range

The lytic activity and specificity of phages was determined by screening each against different host bacterium strains using the standard double agar overlay method previously described. Serial dilutions of each phage stock were prepared and spotted on *S*. Typhi to obtain a concentration where plaques could be observed. Dilutions where plaques could be distinguished were used to spot phages on *S*. Typhi CT18, *S*. Typhi Ty2, *S*. Typhi Ty2 ΔVi (Vi capsule knockout mutant Δ*tviB*), *S*. Typhi Δ*fliC* (flagellate knockout mutant), *Salmonella* enterica serovar Paratyphi A (ATCC: 9150), *Salmonella* Choleraesuis (ATCC: 13312), *Salmonella* Enteritidis (ATCC: 13076), *Salmonella* Newport (ATCC: 6962), *Salmonella* Saintpaul (clinical isolate), *Salmonella* Typhimurium LT2, *Acinetobacter baumannii* (ATCC: 19606), *Escherichia coli* (ATCC: 25922), *Enterobacter cloacae* (ATCC: 13047), *Klebsiella pneumoniae* (clinical isolate obtained from Dhulikhel Hospital, a Kathmandu University hospital), *Morganella morganii* (ATCC: 25830), *Pseudomonas aeruginosa* (ATCC: 27853), *Proteus mirabilis* (ATCC: 29906), *Serratia marcescens* (ATCC: 13880), *Staphylococcus aureus* (ATCC: 25923), *Vibrio cholerae (*ATCC: 14035), and *Yersinia enterocolitica* (ATCC: 9610).

To test diversity and activity spectra of phages across different *S*. Typhi lineages, phage stocks were also tested against 37 *S*. Typhi clinical isolates obtained from a surveillance study performed in the communities where the environmental sampling was performed. These isolates comprise different genotypes circulating in Nepal (S1 Table).

### Statistical analysis

We constructed generalized linear mixed-effect models using the *lme4* package in R to test for differences between sample positivity based on segments of the Kathmandu Valley rivers (upstream, city center, and downstream). Statistical significance was assessed at a % level using a Wald test.

### Whole-genome sequencing

We randomly selected 27 phages from our collection to perform whole genome sequencing. Bacteriophage DNA was extracted using the Phage DNA Isolation Kit (Norgen Biotek Corp., Thorold, ON, Canada) according to the manufacturer’s instructions. The purity and concentration of the DNA were determined using a Qubit 4 fluorometer (Invitrogen, Qubit). Whole genome sequencing was performed using the Illumina Miseq platform (Illumina, San Diego, CA, USA) to generate paired-end reads of 300 bp in length. Sequence data quality was checked using FastQC v0.11.9 to remove low quality reads [18]. We summarized all quality indicators using MultiQC v1.7 [19]. Species identification was confirmed with Kraken2 [20]. Genome assembly was performed using SPAdes Genome Assembler v.3.15.2 and assemblies that resolved as one contig were included in the analysis. Finally, the genomes were annotated using the RAST server (Rapid Annotation using Subsystem Technology) (https://rast.nmpdr.org/rast.cgi) with subsequent manual curation. To determine the family-level classification of the phages, we used VirClust [21] to calculate protein-based hierarchical clustering trees. Whole proteome intergenomic distances were calculated using VICTOR [22]. To resolve genus- and species-level groups, we used VIRIDIC [23] nucleotide-based whole genome distances among the phages (95 and 70% nucleotide identity thresholds, respectively). In addition, the phage proteomes were analyzed using the Phage Classification Tool Set (PHACTS), a computational classification algorithm trained to predict phage lifestyles [24].

### Phylogenetic analysis

The annotation of complete phage genomes was also manually inspected using Geneious version 11.0.12 (Biomatters Ltd) to screen for the presence of phage-related regions containing structural proteins. The complete sequences were analyzed adjusting the direction of nucleotide sequences. Phylogenetic trees were generated using selected concatenated phage signature genes commonly used to understand phage phylogeny. All genomes were screened for the presence of phage signature genes and phylogenetic trees were generated using selected concatenated sequences of genes encoding terminase and tail fiber structures [25–29]. We constructed alignments using the program ClustalW version 2.1 with default parameters. Phylogenetic tree was inferred using the Maximum Likelihood method with RAxML v8.2.10. Bootstrapping was set to 100 replicates and the tree rooted. The resulting phylogenies were visualized and annotated using the iTOL v5 online version [30]. An additional 27 fiber tail protein sequences and 27 terminase protein sequences from GenBank were included in the phylogenetic analysis for comparison. We selected *Caudovirales* representative phage genomes that were matched with our phage collection using IDseq (https://czid.org/). The genomes that matched our sequences with average percent-identity higher than 90% were included in the analysis. The additional 27 bacteriophage genomes selected for study were isolated from five different host genus: *Citrobacter sp*., *Escherichia sp*., *Klebsiella sp*., *Salmonella spp*., and *Shigella sp*., from different geographical locations.

## 3. Results

### Sampling and phage detection

**Fig 1:**
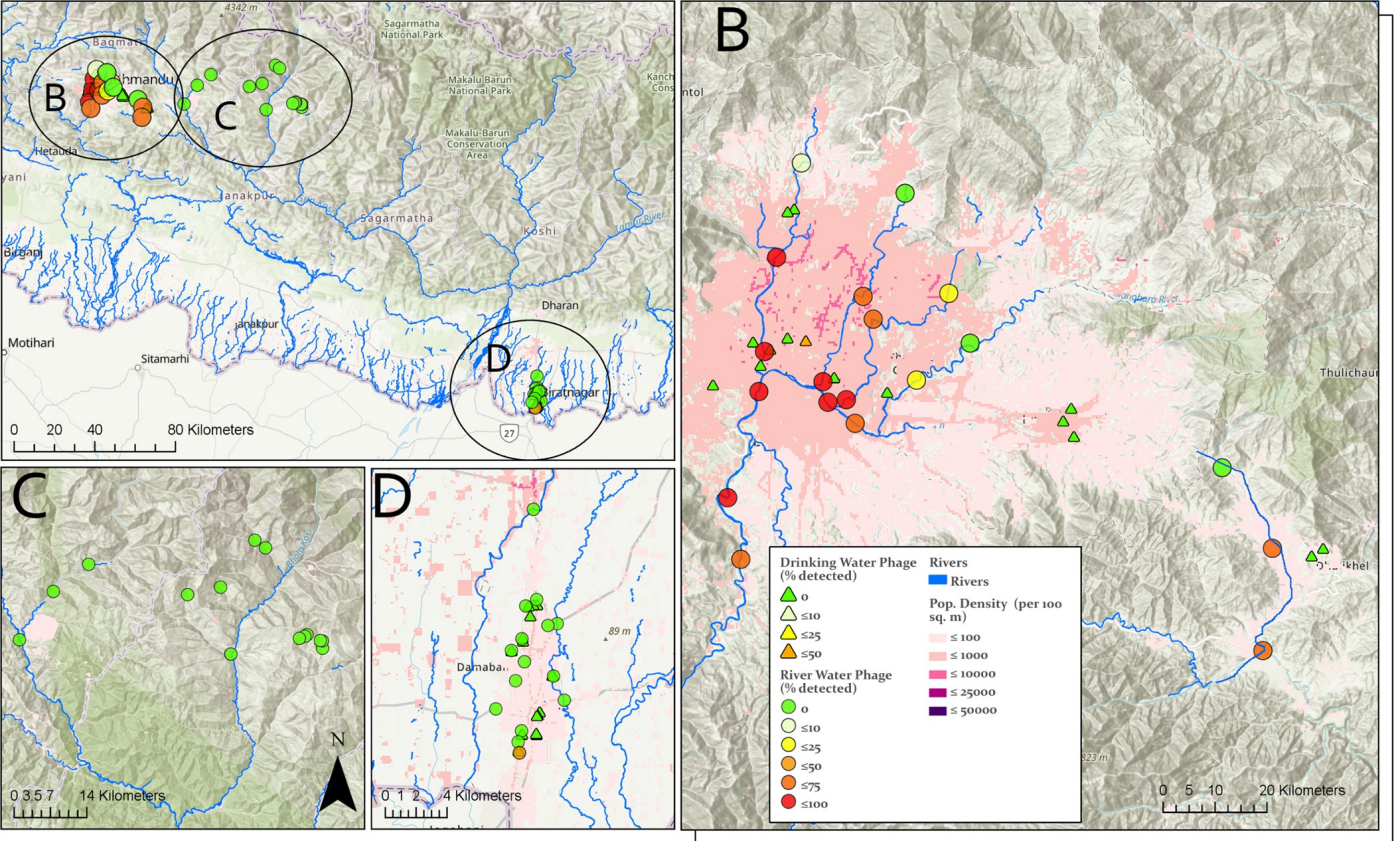
(**a**) Sampling location and detection of *Salmonella* Typhi phages in Nepal. Environmental water sample collection locations and percentage of samples collected with phage detection performed in (**b)** Kathmandu Valley and neighboring Kavrepalanchok district, **(c)** rural Dolakha and (**d**) urban Biratnagar. Topographic maps are overlayed with population density by shades of red. The basemap layer is from Open Street Maps (https://download.geofabrik.de/asia/nepal.html) and was rendered using ArcGIS Pro (https://www.esri.com/en-us/arcgis/products/arcgis-pro/overview).

A total of 428 environmental water samples were collected from four different regions in Nepal (Table 1; Fig 1). Between November 2019 and October 2021, we collected 377 samples from Kathmandu and Kavrepalanchok. These samples included river water (n = 361) from 19 sites and drinking water (n = 16) from 16 households (Fig 1B). *S*. Typhi phages were present in 54.8% (198/361) of the total river samples and 6.3% (1/16) of the drinking water samples. Most samples (72.6%, 193/266) collected within or downstream of the densely populated parts of the city centers contained *S*. Typhi phages, while only 5.3% (5/95) of samples from the sites upstream to these city centers contained these phages. No seasonal pattern was observed for phage detection (S1 Fig).

**Table 1:** *S*. Typhi phage isolation from environmental samples collected in Nepal.

Sampling Site	Type of sample	Sites (n)	Samples (n)	Phage positive samples	Positivity (%)	Dates of sampling
**Kathmandu/Kavre**	River water	19	361	198	54.8%	November 2019- October 2021
	Drinking Water	16	16	1	6.3%	
**Dolakha**	River water	16	16	0	0%	October 2021
**Biratnagar**	River water	7	7	0	0%	May 2021
	River Canal	8	8	1	12.5%	
	Drinking Water	20	20	0	0%	

In September 2021, a total of 16 river water samples were collected from Dolakha (Fig 1C), and none contained *S*. Typhi phages. In May 2022, a total of 35 environmental water samples were also collected from Biratnagar. These samples included river (n = 7), canal (n = 8), and drinking water (n = 20). No *S*. Typhi phages were detected in river or drinking water; one sample from canal water contained *S*. Typhi phages.

### Host range properties

**Fig 2:**
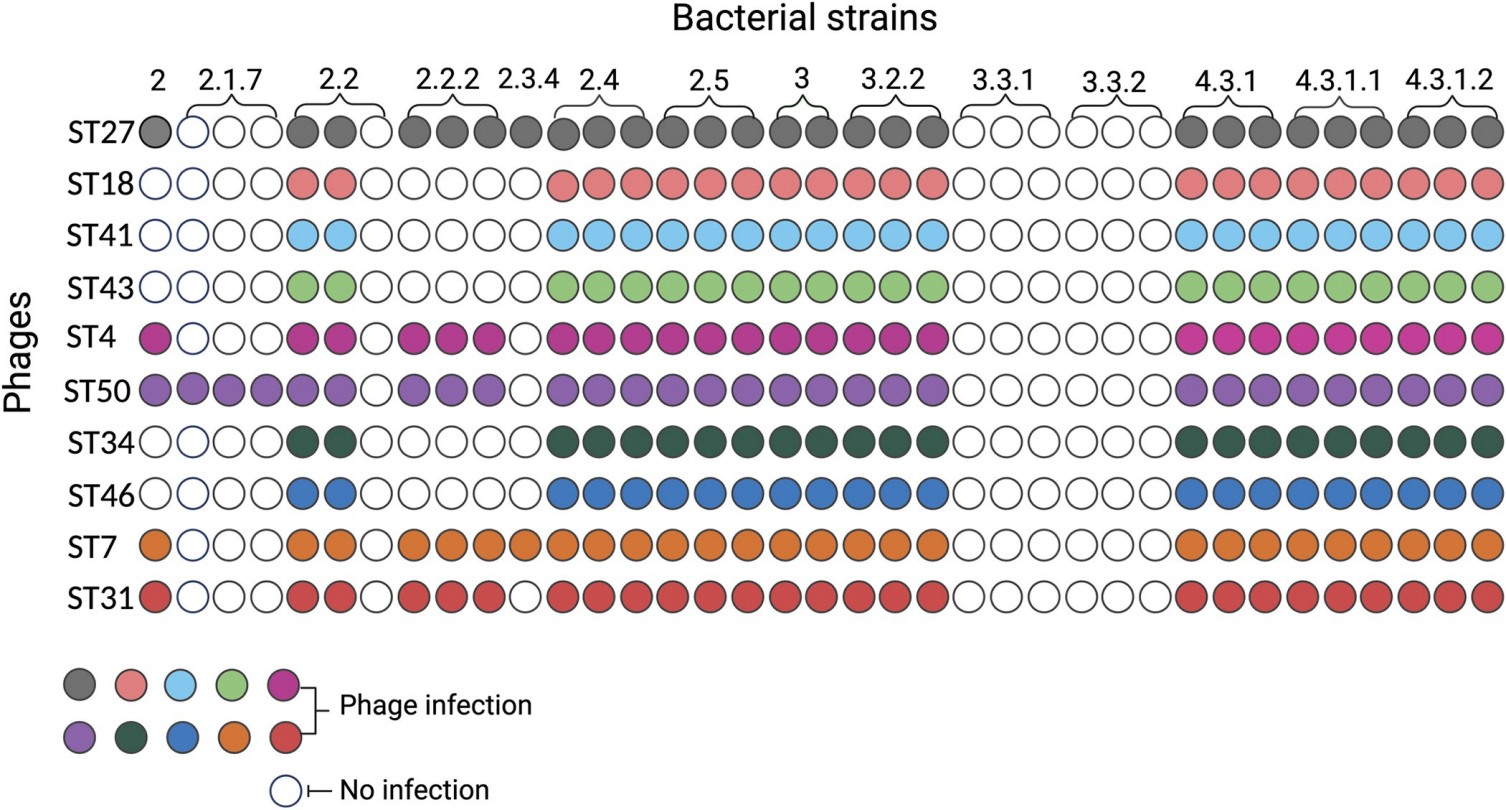
Host range activity of 10 *S*. Typhi phages isolated in our study. *S*. Typhi phages (y-axis) were spotted on *S*. Typhi isolates (x-axis) belonging to different genotypes. Colored circles represent lytic activity, white circles represent no lytic activity.

To evaluate the host range and specificity of *S*. Typhi phages isolated, we tested a total of 50 phages against a panel of relevant strains including additional *Salmonella enterica* serovars (Choleraesuis, Enteritidis, Newport, Paratyphi A, Saintpaul, and Typhimurium) and other bacteria such as *Acinetobacter baumannii*, *Escherichia coli*, *Enterobacter cloacae*, *Klebsiella pneumoniae*, *Morganella morganii*, *Pseudomonas aeruginosa*, *Proteus mirabilis*, *Serratia marcescens*, *Staphylococcus aureus*, *Vibrio cholerae* and *Yersinia enterocolitica*. Host ranges were determined by spotting phage solution onto bacterial lawns. The results show that all 50 phages tested were only capable of infecting *S*. Typhi strains including a flagella knockout *S*. Typhi strain (Δ*fliC*) and could not infect other bacteria. Although *S*. Typhi Δ*fliC* was susceptible to phage infection, no plaques were formed on the Ty2 isogenic Vi-negative *S*. Typhi strain (Δ*tviB*). Additionally, to understand the diversity of the phages collected, we tested the host range activity of 10 phages against a panel of 37 *S*. Typhi strains belonging to 14 different genotypes circulating in Nepal (Fig 2). These *S*. Typhi strains were isolated as part of our comprehensive genomic epidemiological study [31], which involved the sequencing of *S*. Typhi strains obtained from prospective surveillance. The results of our analysis unveiled a total of 17 distinct *S*. Typhi genotypes circulating in Nepal, highlighting the considerable diversity within the *S*. Typhi population in this region. The phages tested were isolated from different sampling sites at different time points and were selected, based on different plaque morphologies. The results showed a broad lytic activity against the *S*. Typhi strains tested. Interestingly, *S*. Typhi strains belonging to lineages 2.1.7, 3.3.1 and 3.3.2 were resistant to the majority of phages tested.

### Characterization of phage genomes

The basic genome metrics of the 27 phage sequences included in this study (host, genome size, GC content, number of predicted ORFs) are provided in Table 2. The analyzed *S*. Typhi phages showed diversity of genome size from 37 to 47 kb, encoding 44 – 73 ORFs. No tRNAs were predicted in the phage genomes. To further investigate genomic characteristics, we conducted a comprehensive analysis using PHACTS to predict the phages’ lifestyle. The results of the PHACTS analysis consistently predicted a lytic lifestyle for all the phages isolated in this study.

**Table 2:** Genome characteristics of 27 *S*. Typhi phages sequenced in our study.

	Phage	Family	Genus	Genome size (bp)	GC %	CDS
1	ST1	*Autographiviridae*	*Teseptimavirus*	38,461	50.9	49
2	ST3	*Autographiviridae*	*Teseptimavirus*	38,461	50.9	48
3	ST10	*Autographiviridae*	*Teseptimavirus*	38,059	51.1	53
4	ST11	*Autographiviridae*	*Teseptimavirus*	38,664	51.1	52
5	ST15	*Autographiviridae*	*Teseptimavirus*	38,351	50.9	49
6	ST16	*Autographiviridae*	*Teseptimavirus*	38,234	50.9	51
7	ST17	*Autographiviridae*	*Teseptimavirus*	38,668	50.9	52
8	ST20	*Autographiviridae*	*Teseptimavirus*	37,644	51.0	44
9	ST21	*Autographiviridae*	*Teseptimavirus*	38,416	51.0	48
10	ST29	*Autographiviridae*	*Teseptimavirus*	39,065	51.0	49
11	ST34	*Autographiviridae*	*Teseptimavirus*	38,880	51.0	48
12	ST35	*Autographiviridae*	*Teseptimavirus*	38,880	51.0	48
13	ST37	*Autographiviridae*	*Teseptimavirus*	38,880	51.0	48
14	ST38	*Autographiviridae*	*Teseptimavirus*	37,743	50.9	48
15	ST43	unclassified *Caudoviricete*	*Roufviru*	45,854	46.2	72
16	ST44	unclassified *Caudoviricete*	*Roufvirus*	45,854	46.2	73
17	ST45	*Autographiviridae*	*Teseptimavirus*	38,351	50.9	50
18	ST46	unclassified *Caudoviricete*	*Roufvirus*	47,596	46.1	76
19	ST53	*Autographiviridae*	*Teseptimavirus*	39,134	50.9	52
20	ST55	*Autographiviridae*	*Teseptimavirus*	39,134	50.9	52
21	ST56	*Autographiviridae*	*Teseptimavirus*	37,877	51.0	50
22	ST57	*Autographiviridae*	*Teseptimavirus*	37,407	51.0	47
23	ST59	*Autographiviridae*	*Teseptimavirus*	39,134	50.9	52
24	ST63	*Autographiviridae*	*Teseptimavirus*	39,134	50.9	52
25	ST64	*Autographiviridae*	*Teseptimavirus*	39,133	50.9	52
26	ST65	unclassified *Caudoviricete*	*Roufvirus*	45,855	46.2	73
27	ST66	*Autographiviridae*	*Teseptimavirus*	39,118	50.9	53

We evaluated relationships among phages using VIRCLUST and VICTOR. VIRCLUST resolved the phages into two distinct family-level units (S2, S3 Figs and S3 Table) on the basis of shared core Protein Super Clusters, and similar clusters were obtained by VICTOR subfamily taxon predictions (based on amino acids and d4 distance formula (S4 Fig). We resolved genus- and species-level units on the basis of whole genome nucleotide similarity using VIRIDIC. Our analysis showed that phages isolated in our study belong to 2 distinct genera and 12 species with intergenomic similarity of 95% and higher. (S5 Fig)

**Fig 3:**
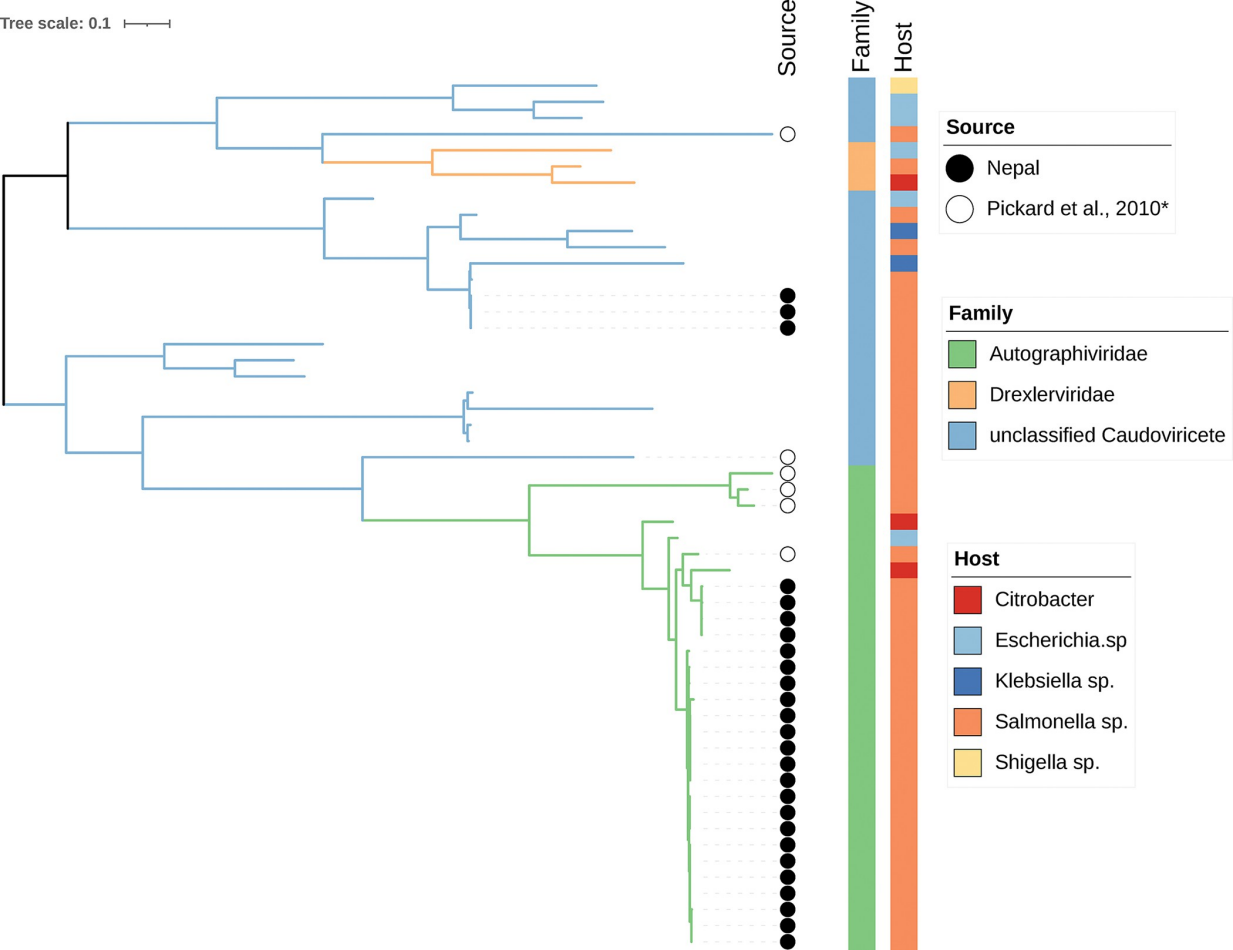
Phylogenetic tree of 27 S. Typhi phages isolated from Nepal and other 27 phages based on two gene sequences (tail fiber and terminase). Tail fiber and terminase large subunit sequences of related phages were downloaded from NCBI. Phages isolated in our study are annotated with a black circle. Pickard et al., 2010 [15]* represents a *S*. Typhi Vi phage collection obtained from Cambridge University, Cambridge, United Kingdom, and originally isolated between the 1930 and 1955. These historical phages are annotated with white circles.

The tail fiber and large terminase subunit protein sequences are commonly used markers for understanding phage phylogeny. The phylogenetic relationship was established using the identified terminase and tail fiber nucleotide sequences. The alignment of all sequences revealed that the *S*. Typhi phages isolated in our study can be categorized into two different viral families, forming three distinct phylogenetic clusters. Specifically, phage sequences classified as members of the *Autographiviridae* family were assigned to the *Teseptimavirus* genus, while the remaining sequences from our collection were attributed to the *Roufvirus* genus. Notably, within the *Teseptimavirus* genus, we identified two distinct subgroups. (Fig 3). To better understand the phylogenetic and evolutionary relationship of the phages from our collection, we compared their genomes with previously sequenced phages deposited in GenBank. A total of 27 representative phage genomes belonging to five different families were included and our analysis identified at least 9 distinct clusters. Our findings showed that phages from our collection did not cluster closely with any other phage previously described.

## 4. Discussion

In typhoid-endemic communities in and around the Kathmandu Valley of Nepal, we found that *S*. Typhi-specific bacteriophages were widely present in multiple rivers that were contaminated with sewage and readily detectable by small-volume water sampling. By contrast, in a rural and an urban community where typhoid is uncommon, and no visible waste contamination was observed, *S*. Typhi bacteriophages were rarely detected. Taken together, these findings support the premise of phage-based assays as a simple and low-cost tool for environmental surveillance for typhoid, which could be useful for identifying settings to prioritize for vaccination and clean water and sanitation interventions or monitoring their impact after introduction.

Our findings are consistent with the epidemiological data obtained from previous studies we conducted in Nepal. Our prospective surveillance study conducted from 2016 to 2019 in Kathmandu and Kavrepalanchok revealed a high burden of typhoid fever [7]. Additionally, these studies have identified a positive association between population density and typhoid incidence, with higher rates in urban compared with rural communities [16,32]. Further studies are needed across gradients of typhoid endemicity to characterize the relationship between phage abundance in environmental samples and community typhoid incidence.

Although the detection of *S*. Typhi DNA in municipal drinking water in Kathmandu has been previously reported [33] in our study *S*. Typhi phages were detected in only one drinking water sample. These findings are consistent with our previous work conducted in Kathmandu and Kavrepalanchok, Nepal [34]. We collected drinking water samples and performed quantitative real-time PCR to detected DNA sequences specific for *S*. Typhi. Three hundred and eighty water samples were collected from a randomly selected subset of households and seven samples tested positive for *S*. Typhi [34]

Our pilot study aimed to generate an easily reproducible approach to be performed in other sites to investigate the role of phages in shaping epidemiologic patterns of typhoid transmission. This will provide an opportunity to elucidate potential differences and similarities in the distribution and abundance of *S*. Typhi in different regions. Our results emphasize the feasibility of employing an attenuated *S*. Typhi Ty2 strain (BRD948) in our approach, which is recognized as safe for use within containment level two laboratories. Based on our findings, we recommend the continued use of the BRD948 strain in future research. It’s worth noting that, even when working with attenuated strains, these assays still require access to microbiological laboratory facilities, which may not be readily available in certain regions. However, our research demonstrates that phage detection is a cheaper and simpler process than the common methodologies used for detecting bacterial DNA from water samples. Our simplified approach requires minimal supplies, including media for bacterial growth, an incubator, a centrifuge, pipettes, petri dishes, tubes, syringes, and syringe filters. The total cost is <$2 for each sample and given the minimal resources required can be done in labs with very basic infrastructure and equipment.

Our results showed that phages isolated in our study are highly specific to *S*. Typhi isolates. As *Salmonella* consists of more than 2,500 serovars, infection efficacy and specificity are indispensable requirements to the establishment of phage environmental surveillance as a marker for typhoid burden. To further investigate patterns of host infectivity, we tested phages of our collection against Vi capsule-negative and flagellum-negative *S*. Typhi strains. Phages isolated in our study did not infect Vi-capsule mutant strain, demonstrating the requirement of Vi capsule expression for infectivity, as characterized in earlier work [35] We also evaluated the ability of phages to infect different *S*. Typhi genotypes. Interestingly, phages from our collection were unable to infect lineages 3.3.1 and 3.3.2. Our findings suggest that the observed variability in phage susceptibility among *S*. Typhi lineages may be attributed to natural variation in Vi or presence of phage defense strategies preventing infection or subsequent cell lysis. Bacteria can evade phage infection by several mechanisms, including accumulating escape mutations in the receptor, acquiring phage inhibitory proteins, or directly modifying the receptor and using CRISPR-Cas system as a phage defense mechanism [36]. Further studies are needed to elucidate the specific host defense mechanisms that *S*. Typhi uses to protect against phage predation, and whether the acquisition of these defense mechanisms drives selection for certain lineages containing them.

Phages from our collection were sequenced and analyzed on the base of genetic similarity to each other and to other phage genomes previously described. Sequence comparison allowed for clear discrimination of distinct clusters. In addition, phylogenetic analysis showed that phages isolated from Nepal clustered independently from other phages included in our analysis; however, a limited number of *S*. Typhi-specific phages have been sequenced and reported. Further studies investigating *S*. Typhi phages in neighboring typhoid-endemic countries and in other regions of the world could further the position of the phages we identified within the global diversity of *S*. Typhi phages.

A series of studies from the cholera field revealed that *Vibrio cholerae* phages exhibit predator-prey dynamics that shape cholera abundance in water, potentially altering seasonal disease patterns [37]. A study from Kolkata in 1930 found a similar inverse, seasonal relationship between *S*. Typhi phage abundance and clinical cases, which could support a similar disease dynamic [13]. In our study, we did not observe a clear seasonal pattern for phage detection, despite the known variations in rainfall intensity and timing during the rainy season in Nepal. Our sampling period, which spanned the height of COVID-19 transmission and related lockdowns, may not have enabled us to accurately detect the influence of seasonality on phage detection. Further studies are needed to understand the potential role of *S*. Typhi phages in shaping the ecology of endemic typhoid transmission. Additionally, while we recognize the importance of considering other factors, such as rainfall patterns and population movements such as holidays and traditional celebrations, our study primarily focused on assessing the presence of *S*. Typhi phages in environmental water samples. It’s important to note that the vast majority of urban households in the region lack connections to the sewer system, and wastewater treatment infrastructure is underdeveloped [38], further highlighting the complexity of assessing the impact of wastewater treatment facilities on phage presence. Therefore, while these factors are relevant, their influence on phage detection may be limited, and further research in these areas is needed.

The results of this study should be interpreted within the context of several limitations. First, we collected a limited number of drinking water samples, and although we observed a low positivity rate, this doesn’t mean that drinking water is not an important transmission route for typhoid in this setting. It is possible that drinking water contamination is more sporadic and wasn’t detected in our limited sampling. Additionally, the scarcity of drinking water in the Kathmandu Valley has led to increased reliance on drinking water that is brought in by large tanker trucks from outside the valley, where typhoid incidence (and contamination risk) is likely lower. Second, the presence of phages in river water reflects their presence in sewage, which is helpful for monitoring burden in the population, but does not directly implicate river water in the transmission pathway. Third, further studies are needed to validate our assay quantitatively in order to specify a limit of detection and sensitivity against a gold standard, such as PCR-based detection methods [9]. Fourth, further studies are required correlating frequency, level, geography, and timing of detections of different phages with estimates of disease burden.

Our study highlights the strength of a simplified phage plaque assay as a low-cost tool to identify communities where typhoid is endemic. The high abundance of phages in river water suggest that this could be a scalable and more sensitive alternative to molecular methods for environmental surveillance for typhoid. The findings of our study will inform potential widely implementation of phage environmental surveillance to generate data to support vaccine introduction, monitor intervention strategies and improve understanding of the emergence and transmission of *S*. Typhi in high-risk settings.

## Supporting information

S1 Table Bacterial strains used in this study. (DOCX)

S2 Table The main characteristics of river and drinking water samples collected in the study area. (XLSX)

S3 Table Summary of phage genomics and protein clusters statistics generated by Virclust. (XLSM)

S1 Fig Temporal variation in phage detection in Kathmandu, Nepal during the study period. (TIF)

S2 Fig Heatmap showing pairwise intergenomic similarities (%) of *S*. Typhi isolated from Nepal. The numbers and colors indicate similarity between the phage genomes from none or lower (red) to high (dark red).
(TIF)

S3 Fig VirClust hierarchical clustering of the *S*. Typhi isolated from Nepal, based on intergenomic distances calculated using the protein cluster content. The genome clustering was performed based on PCs. The resulting tree was split into VGCs using a 0.9 intergenomic distance threshold. The visual components are described further. **1**. Hierarchical tree calculated using PC-based intergenomic distances. **2**. Silhouette width, color-coded in a range from −1 (red) to 1 (green). **3**. VGC ID. **4**. Heatmap representation of the PC distribution in the viral genomes. Rows are represented by individual viral genomes. Columns are represented by individual PCs. The ID of each PC can be read at the bottom of the heatmap. Colors encode the number of each PC per genome, with white signifying the PC absence, and the other colors signifying various degrees of replication. **5**. Viral genome-specific statistics: genome length, the proportion of PC shared (dark grey) with any other genomes in the dataset, reported to the total PCs in the genome (light grey bar), the proportion of PC shared in its own VGC, the proportion of PCs shared only in its own VGC, the proportion of PCs shared also outside its own VGC, and the proportion of PC shared only outside own VGC. **6**. *S*. Typhi phage name.
(TIF)

S4 Fig Phylogenetic tree of *S*. Typhi isolated from Nepal. All pairwise comparisons of the nucleotide sequences were conducted using the Genome-BLAST Distance Phylogeny (GBDP) method under settings recommended for prokaryotic viruses using VICTOR software. The resulting intergenomic distances were used to infer a balanced minimum evolution tree with branch support via FASTME including SPR postprocessing. Branch support was inferred from 100 pseudo-bootstrap replicates each. Branches with bootstrap values below 50 were collapsed and the bootstrap values equal to or above 50 are shown on the remainder of the tree branches.
(TIF)

S5 Fig Nucleotide-based intergenomic similarities of *S*. Typhi isolated from Nepal, using VIRIDIC. A heatmap of hierarchical clustering of the intergenomic similarity values was generated and given as percentage values (right half, blue-green heatmap). Each genome pair is represented by three values (left half), where the top and bottom represent the aligned genome fraction for the genome in the row and column, respectively. The middle value represents the genome length ratio for each genome pair.
(TIF)

## References

[1] Kumar A, Kumar A. Antibiotic resistome of Salmonella typhi: molecular determinants for the emergence of drug resistance. Front Med. 2021;15: 693–703. doi: 10.1007/s11684-020-0777-6 34085183

[2] Stanaway JD, Reiner RC, Blacker BF, Goldberg EM, Khalil IA, Troeger CE, et al. The global burden of typhoid and paratyphoid fevers: a systematic analysis for the Global Burden of Disease Study 2017. Lancet Infect Dis. 2019;19: 369–381. doi: 10.1016/S1473-3099(18)30685-6 30792131 PMC6437314

[3] Andrews JR, Barkume C, Yu AT, Saha SK, Qamar FN, Garrett D, et al. Integrating Facility-Based Surveillance with Healthcare Utilization Surveys to Estimate Enteric Fever Incidence: Methods and Challenges. Journal of Infectious Diseases. 2018;218: S268–S276. doi: 10.1093/infdis/jiy494 30184162 PMC6226762

[4] Qamar FN, Yousafzai MT, Dehraj IF, Shakoor S, Irfan S, Hotwani A, et al. Antimicrobial Resistance in Typhoidal Salmonella: Surveillance for Enteric Fever in Asia Project, 2016–2019. Clinical Infectious Diseases. 2020;71: S276–S284. doi: 10.1093/cid/ciaa1323 33258934 PMC7705872

[5] Date K, Shimpi R, Luby S, N R, Haldar P, Katkar A, et al. Decision Making and Implementation of the First Public Sector Introduction of Typhoid Conjugate Vaccine-Navi Mumbai, India, 2018. Clin Infect Dis. 2020;71: S172–S178. doi: 10.1093/cid/ciaa597 32725235 PMC7420725

[6] World Health Organization. Typhoid vaccines: WHO position paper, March 2018 – Recommendations. Vaccine. 2019;37: 214–216. doi: 10.1016/j.vaccine.2018.04.022 29661581

[7] Garrett DO, Longley AT, Aiemjoy K, Yousafzai MT, Hemlock C, Yu AT, et al. Incidence of typhoid and paratyphoid fever in Bangladesh, Nepal, and Pakistan: results of the Surveillance for Enteric Fever in Asia Project. Lancet Glob Health. 2022;10: e978–e988. doi: 10.1016/S2214-109X(22)00119-X 35714648 PMC9210262

[8] Andrews JR, Yu AT, Saha S, Shakya J, Aiemjoy K, Horng L, et al. Environmental Surveillance as a Tool for Identifying High-risk Settings for Typhoid Transmission. Clin Infect Dis. 2020;71: S71–S78. doi: 10.1093/cid/ciaa513 32725227 PMC7446943

[9] Matrajt G, Lillis L, Meschke JS. Review of Methods Suitable for Environmental Surveillance of Salmonella Typhi and Paratyphi. Clinical Infectious Diseases. 2020;71: S79–S83. doi: 10.1093/cid/ciaa487 32725228 PMC7388719

[10] Uzzell CB, Troman CM, Rigby J, Mohan VR, John J, Abraham D, et al. Environmental surveillance for Salmonella Typhi as a tool to estimate the incidence of typhoid fever in low-income populations. medRxiv. 2021. doi: 10.1101/2021.05.21.21257547

[11] Hagedorn BL, Zhou NA, Fagnant-Sperati CS, Shirai JH, Gauld J, Wang Y, et al. The cost of building an environmental surveillance system for typhoid. medRxiv. 2021; 2021.07.12.21260395. doi: 10.1101/2021.07.12.21260395

[12] Rogovski P, Cadamuro RD, da Silva R, de Souza EB, Bonatto C, Viancelli A, et al. Uses of Bacteriophages as Bacterial Control Tools and Environmental Safety Indicators. Front Microbiol. 2021;12. doi: 10.3389/fmicb.2021.793135 34917066 PMC8670004

[13] Pasricha CL, de Monte AJ, Gupta SK. Seasonal Variations of Typhoid Bacteriophage in Natural Waters and in Man, in Calcutta during the Year 1930. Ind Med Gaz. 1931;66: 549–550. 29009946 PMC5185636

[14] Tacket CO, Sztein MB, Losonsky GA, Wasserman SS, Nataro JP, Edelman R, et al. Safety of live oral Salmonella typhi vaccine strains with deletions in htrA and aroC aroD and immune response in humans. Infect Immun. 1997;65: 452–456. doi: 10.1128/iai.65.2.452-456.1997 9009296 PMC174616

[15] Pickard D, Toribio AL, Petty NK, Van Tonder A, Yu L, Goulding D, et al. A conserved acetyl esterase domain targets diverse bacteriophages to the Vi capsular receptor of Salmonella enterica serovar typhi. J Bacteriol. 2010;192: 5746–5754. doi: 10.1128/JB.00659-10 20817773 PMC2953684

[16] Andrews JR, Vaidya K, Bern C, Tamrakar D, Wen S, Madhup S, et al. High Rates of Enteric Fever Diagnosis and Lower Burden of Culture-Confirmed Disease in Peri-urban and Rural Nepal. J Infect Dis. 2018;218: S214–S221. doi: 10.1093/infdis/jix221 28961918 PMC6226739

[17] Kropinski AM, Mazzocco A, Waddell TE, Lingohr E, Johnson RP. Enumeration of Bacteriophages by Double Agar Overlay Plaque Assay. In: Clokie MRJ, Kropinski AM, editors. Bacteriophages: Methods and Protocols, Volume 1: Isolation, Characterization, and Interactions. Totowa, NJ: Humana Press; 2009. pp. 69–76. doi: 10.1007/978-1-60327-164-6_7 19066811

[18] Andrews S. Babraham Bioinformatics - FastQC A Quality Control tool for High Throughput Sequence Data. Soil. 1973. doi: 10.1016/0038-0717(73)90093-X

[19] Ewels P, Magnusson M, Lundin S, Käller M. MultiQC: Summarize analysis results for multiple tools and samples in a single report. Bioinformatics. 2016. doi: 10.1093/bioinformatics/btw354 27312411 PMC5039924

[20] Wood DE, Lu J, Langmead B. Improved metagenomic analysis with Kraken 2. Genome Biol. 2019. doi: 10.1186/s13059-019-1891-0 31779668 PMC6883579

[21] Moraru C. VirClust—A Tool for Hierarchical Clustering, Core Protein Detection and Annotation of (Prokaryotic) Viruses. Viruses. 2023;15. doi: 10.3390/v15041007 37112988 PMC10143988

[22] Meier-Kolthoff JP, Göker M. VICTOR: genome-based phylogeny and classification of prokaryotic viruses. Bioinformatics. 2017;33: 3396–3404. doi: 10.1093/bioinformatics/btx440 29036289 PMC5860169

[23] Moraru C, Varsani A, Kropinski AM. VIRIDIC—A novel tool to calculate the intergenomic similarities of prokaryote-infecting viruses. Viruses. 2020;12. doi: 10.3390/v12111268 33172115 PMC7694805

[24] McNair K, Bailey BA, Edwards RA. PHACTS, a computational approach to classifying the lifestyle of phages. Bioinformatics. 2012;28: 614–618. doi: 10.1093/bioinformatics/bts014 22238260 PMC3289917

[25] Ariff A, Wise MJ, Kahler CM, Tay CY, Peters F, Perkins TT, et al. Novel Moraxella catarrhalis prophages display hyperconserved non-structural genes despite their genomic diversity. BMC Genomics. 2015;16. doi: 10.1186/s12864-015-2104-1 26497500 PMC4619438

[26] Sritha KS, Bhat SG. Genomics of Salmonella phage ΦStp1: candidate bacteriophage for biocontrol. Virus Genes. 2018;54: 311–318. doi: 10.1007/s11262-018-1538-3 29478159

[27] Sørensen PE, Van Den Broeck W, Kiil K, Jasinskyte D, Moodley A, Garmyn A, et al. New insights into the biodiversity of coliphages in the intestine of poultry. Sci Rep. 2020;10. doi: 10.1038/s41598-020-72177-2 32939020 PMC7494930

[28] Amarillas L, Chaidez C, González-Robles A, León-Félix J. Complete genome sequence of new bacteriophage phiE142, which causes simultaneously lysis of multidrug-resistant Escherichia coli O157: H7 and Salmonella enterica. Stand Genomic Sci. 2016;11. doi: 10.1186/s40793-016-0211-5 27999624 PMC5154165

[29] Colavecchio A, D’Souza Y, Tompkins E, Jeukens J, Freschi L, Emond-Rheault JG, et al. Prophage integrase typing is a useful indicator of genomic diversity in Salmonella enterica. Front Microbiol. 2017;8. doi: 10.3389/fmicb.2017.01283 28740489 PMC5502288

[30] Letunic I, Bork P. Interactive Tree of Life (iTOL) v4: Recent updates and new developments. Nucleic Acids Res. 2019. doi: 10.1093/nar/gkz239 30931475 PMC6602468

[31] da Silva KE, Tanmoy AM, Pragasam AK, Iqbal J, Sajib MSI, Mutreja A, et al. The international and intercontinental spread and expansion of antimicrobial-resistant Salmonella Typhi: a genomic epidemiology study. Lancet Microbe. 2022;3: e567–e577. doi: 10.1016/S2666-5247(22)00093-3 35750070 PMC9329132

[32] Tamrakar D, Vaidya K, Yu AT, Aiemjoy K, Naga SR, Cao Y, et al. Spatial Heterogeneity of Enteric Fever in 2 Diverse Communities in Nepal. Clinical Infectious Diseases. 2020;71: S205–S213. doi: 10.1093/cid/ciaa1319 33258932 PMC7705881

[33] Karkey A, Jombart T, Walker AW, Thompson CN, Torres A, Dongol S, et al. The Ecological Dynamics of Fecal Contamination and Salmonella Typhi and Salmonella Paratyphi A in Municipal Kathmandu Drinking Water. PLoS Negl Trop Dis. 2016;10: e0004346. Available: doi: 10.1371/journal.pntd.0004346 26735696 PMC4703202

[34] LeBoa C, Shrestha S, Shakya J, Naga SR, Shrestha S, Shakya M, et al. Environmental surveillance for typhoidal &lt;em&gt;Salmonellas&lt;/em&gt; in household and surface waters in Nepal identifies potential transmission pathways. medRxiv. 2023; 2023.05.02.23289369. doi: 10.1101/2023.05.02.23289369 PMC10615262 37851667

[35] Derek P, Luisa TA, PN K, Andries van T, Lu Y, David G, et al. A Conserved Acetyl Esterase Domain Targets Diverse Bacteriophages to the Vi Capsular Receptor of Salmonella enterica Serovar Typhi. J Bacteriol. 2010;192: 5746–5754. doi: 10.1128/JB.00659-10 20817773 PMC2953684

[36] Shabbir MAB, Hao H, Shabbir MZ, Wu Q, Sattar A, Yuan Z. Bacteria vs. Bacteriophages: Parallel Evolution of Immune Arsenals. Front Microbiol. 2016;7. doi: 10.3389/fmicb.2016.01292 27582740 PMC4987407

[37] Faruque SM, Bin Naser I, Islam MJ, Faruque ASG, Ghosh AN, Nair GB, et al. Seasonal epidemics of cholera inversely correlate with the prevalence of environmental cholera phages. Proceedings of the National Academy of Sciences. 2005;102: 1702–1707. doi: 10.1073/pnas.0408992102 15653771 PMC547864

[38] Adhikary S, Sharma SP. In Pursuit of Safe Sanitation Services: Governing Fecal Sludge Management in Nepal. 2021.

