# Supplementary material for: Detection of Salmonella Typhi bacteriophages in surface waters as a scalable approach to environmental surveillance
Source: PLoS Negl Trop Dis. 2024 Feb 8;18(2):e0011912. doi: 10.1371/journal.pntd.0011912 (PMC10852241; doi:10.1371/journal.pntd.0011912)
Supplement: S1 Table — (DOCX) [file pntd.0011912.s001.docx]

**Table S1**. Bacterial strains used in this study.

| **Strain** | **Description** | **Method** | **Source** |
| --- | --- | --- | --- |
| *S.* Typhi 27900168 | Clinical isolate | Phage screening/propagation | This study |
| *S*. Typhi Ty2 | Wild type | Phage screening/propagation | Wen et al., 2003 [1] |
| *S*. Typhi BRD948 | *aro*C, *aro*D and HtrA deletions | Propagation | Tacket et al., 1997 [7] |
| *S*. Typhi CT18 | Wild type | Cross-infectivity testing | Parkhill et al., 2001 [3] |
| *S*. Typhi Δ*tviB* | Vi-negative strain | Cross-infectivity testing | Pickard et al., 2008 [4] |
| *S*. Typhi Δ*fliC* | Aflagellated strain | Cross-infectivity testing | Shreiber et al, 2015 [5] |
| *S*. Typhi 27900066 | Genotype 2 | Lineage host range | Silva et al., 2022 [6] |
| *S*. Typhi 27801078 | Genotype 2.1.7 | Lineage host range | Silva et al., 2022 [6] |
| *S*. Typhi MD-2761 | Genotype 2.1.7 | Lineage host range | Silva et al., 2022 [6] |
| *S*. Typhi MD-704 | Genotype 2.1.7 | Lineage host range | Silva et al., 2022 [6] |
| *S*. Typhi 20600116 | Genotype 2.2 | Lineage host range | Silva et al., 2022 [6] |
| *S*. Typhi 20600128 | Genotype 2.2 | Lineage host range | Silva et al., 2022 [6] |
| *S*. Typhi 21300017 | Genotype 2.2 | Lineage host range | Silva et al., 2022 [6] |
| *S*. Typhi 27500064 | Genotype 2.2.2 | Lineage host range | Silva et al., 2022 [6] |
| *S*. Typhi 27801030 | Genotype 2.2.2 | Lineage host range | Silva et al., 2022 [6] |
| *S*. Typhi 27801042 | Genotype 2.2.2 | Lineage host range | Silva et al., 2022 [6] |
| *S*. Typhi 21300122 | Genotype 2.3.4 | Lineage host range | Silva et al., 2022 [6] |
| *S*. Typhi 22000064 | Genotype 2.4 | Lineage host range | Silva et al., 2022 [6] |
| *S*. Typhi 22000091 | Genotype 2.4 | Lineage host range | Silva et al., 2022 [6] |
| *S*. Typhi 22000138 | Genotype 2.4 | Lineage host range | Silva et al., 2022 [6] |
| *S*. Typhi 26700414 | Genotype 2.5 | Lineage host range | Silva et al., 2022 [6] |
| *S*. Typhi 27500035 | Genotype 2.5 | Lineage host range | Silva et al., 2022 [6] |
| *S*. Typhi 27500036 | Genotype 2.5 | Lineage host range | Silva et al., 2022 [6] |
| *S*. Typhi 21300032 | Genotype 3 | Lineage host range | Silva et al., 2022 [6] |
| *S*. Typhi PD-599 | Genotype 3 | Lineage host range | Silva et al., 2022 [6] |
| *S*. Typhi 20600120 | Genotype 3.2.2 | Lineage host range | Silva et al., 2022 [6] |
| *S*. Typhi 22000115 | Genotype 3.2.2 | Lineage host range | Silva et al., 2022 [6] |
| *S*. Typhi 23900002 | Genotype 3.2.2 | Lineage host range | Silva et al., 2022 [6] |
| *S*. Typhi 24300004 | Genotype 3.3.1 | Lineage host range | Silva et al., 2022 [6] |
| *S*. Typhi 25000001 | Genotype 3.3.1 | Lineage host range | Silva et al., 2022 [6] |
| *S*. Typhi 25000071 | Genotype 3.3.1 | Lineage host range | Silva et al., 2022 [6] |
| *S*. Typhi 20600011 | Genotype 3.3.2 | Lineage host range | Silva et al., 2022 [6] |
| *S*. Typhi 20600123 | Genotype 3.3.2 | Lineage host range | Silva et al., 2022 [6] |
| *S*. Typhi 21300160 | Genotype 3.3.2 | Lineage host range | Silva et al., 2022 [6] |
| *S*. Typhi 25000173 | Genotype 4.3.1 | Lineage host range | Silva et al., 2022 [6] |
| *S*. Typhi 25000175 | Genotype 4.3.1 | Lineage host range | Silva et al., 2022 [6] |
| *S*. Typhi 25000182 | Genotype 4.3.1 | Lineage host range | Silva et al., 2022 [6] |
| *S*. Typhi 23900033 | Genotype 4.3.1.1 | Lineage host range | Silva et al., 2022 [6] |
| *S*. Typhi 25000179 | Genotype 4.3.1.1 | Lineage host range | Silva et al., 2022 [6] |
| *S*. Typhi 25000449 | Genotype 4.3.1.1 | Lineage host range | Silva et al., 2022 [6] |
| *S*. Typhi 21300151 | Genotype 4.3.1.2 | Lineage host range | Silva et al., 2022 [6] |
| *S*. Typhi 21300159 | Genotype 4.3.1.2 | Lineage host range | Silva et al., 2022 [6] |
| *S*. Typhi 21300164 | Genotype 4.3.1.2 | Lineage host range | Silva et al., 2022 [6] |
| *S*. Paratyphi A 27900164 | Clinical isolate | Cross-infectivity testing | This study |
| *S*. Paratyphi A 9150 | Wild type | Cross-infectivity testing | ATCC |
| *S.* Choleraesuis | Wild type | Cross-infectivity testing | ATCC |
| *S.* Enteritidis | Wild type | Cross-infectivity testing | ATCC |
| *S.* Newport | Wild type | Cross-infectivity testing | ATCC |
| *S.* Saintpaul | Clinical isolate | Cross-infectivity testing | Silva et al., 2022 [6] |
| *S.* Typhimurium LT2 | Wild type | Cross-infectivity testing | McClelland et al., 2001 [2] |
| *A. baumannii* | Wild type | Cross-infectivity testing | ATCC |
| *E. coli* 25922 | Wild type | Cross-infectivity testing | ATCC |
| *E. coaclae* | Wild type | Cross-infectivity testing | ATCC |
| *K. pneumoniae* | Clinical isolate | Cross-infectivity testing | This study |
| *M. morganii* | Wild type | Cross-infectivity testing | ATCC |
| *P. aeruginosa* 27853 | Wild type | Cross-infectivity testing | ATCC |
| *P. mirabilis* | Wild type | Cross-infectivity testing | ATCC |
| *S. marcescens* | Wild type | Cross-infectivity testing | ATCC |
| *S. aureus* 25923 | Wild type | Cross-infectivity testing | ATCC |
| *V. cholerae* | Wild type | Cross-infectivity testing | ATCC |
| *Y. enterolitica* | Wild type | Cross-infectivity testing | ATCC |

ATCC – American Type Culture Collection.

**References**

1. Wen D, Shian-Ren L, Guy P, F. MG, J. RD, Valerie B, et al. Comparative Genomics of Salmonellaenterica Serovar Typhi Strains Ty2 and CT18. J Bacteriol [Internet]. 2003 Apr 1;185(7):2330–7. Available from: https://doi.org/10.1128/JB.185.7.2330-2337.2003

2. McClelland M, Sanderson KE, Spieth J, Clifton SW, Latreille P, Courtney L, et al. Complete genome sequence of Salmonella enterica serovar Typhimurium LT2. Nature [Internet]. 2001;413(6858):852–6. Available from: https://doi.org/10.1038/35101614

3. Parkhill J, Dougan G, James KD, Thomson NR, Pickard D, Wain J, et al. Complete genome sequence of a multiple drug resistant Salmonella enterica serovar Typhi CT18. Nature [Internet]. 2001;413(6858):848–52. Available from: https://doi.org/10.1038/35101607

4. Pickard D, Thomson NR, Baker S, Wain J, Pardo M, Goulding D, et al. Molecular characterization of the Salmonella enterica serovar typhi Vi-typing bacteriophage E1. J Bacteriol. 2008;190(7):2580–7.

5. Schreiber F, Kay S, Frankel G, Clare S, Goulding D, van de Vosse E, et al. The Hd, Hj, and Hz66 flagella variants of Salmonella enterica serovar Typhi modify host responses and cellular interactions. Sci Rep [Internet]. 2015;5(1):7947. Available from: https://doi.org/10.1038/srep07947

6. da Silva KE, Tanmoy AM, Pragasam AK, Iqbal J, Sajib MSI, Mutreja A, et al. The international and intercontinental spread and expansion of antimicrobial-resistant <em>Salmonella</em> Typhi: a genomic epidemiology study. The Lancet Microbe [Internet]. 2022 Aug 1;3(8):e567–77. Available from: https://doi.org/10.1016/S2666-5247(22)00093-3

7. Tacket CO, Sztein MB, Losonsky GA, Wasserman SS, Nataro JP, Edelman R, et al. Safety of live oral Salmonella typhi vaccine strains with deletions in htrA and aroC aroD and immune response in humans. Infect Immun [Internet]. 1997 Feb 1;65(2):452–6. Available from: https://doi.org/10.1128/iai.65.2.452-456.1997
