# Supplementary figures and images for: Detection of Salmonella Typhi bacteriophages in surface waters as a scalable approach to environmental surveillance
Source: PLoS Negl Trop Dis. 2024 Feb 8;18(2):e0011912. doi: 10.1371/journal.pntd.0011912 (PMC10852241; doi:10.1371/journal.pntd.0011912)

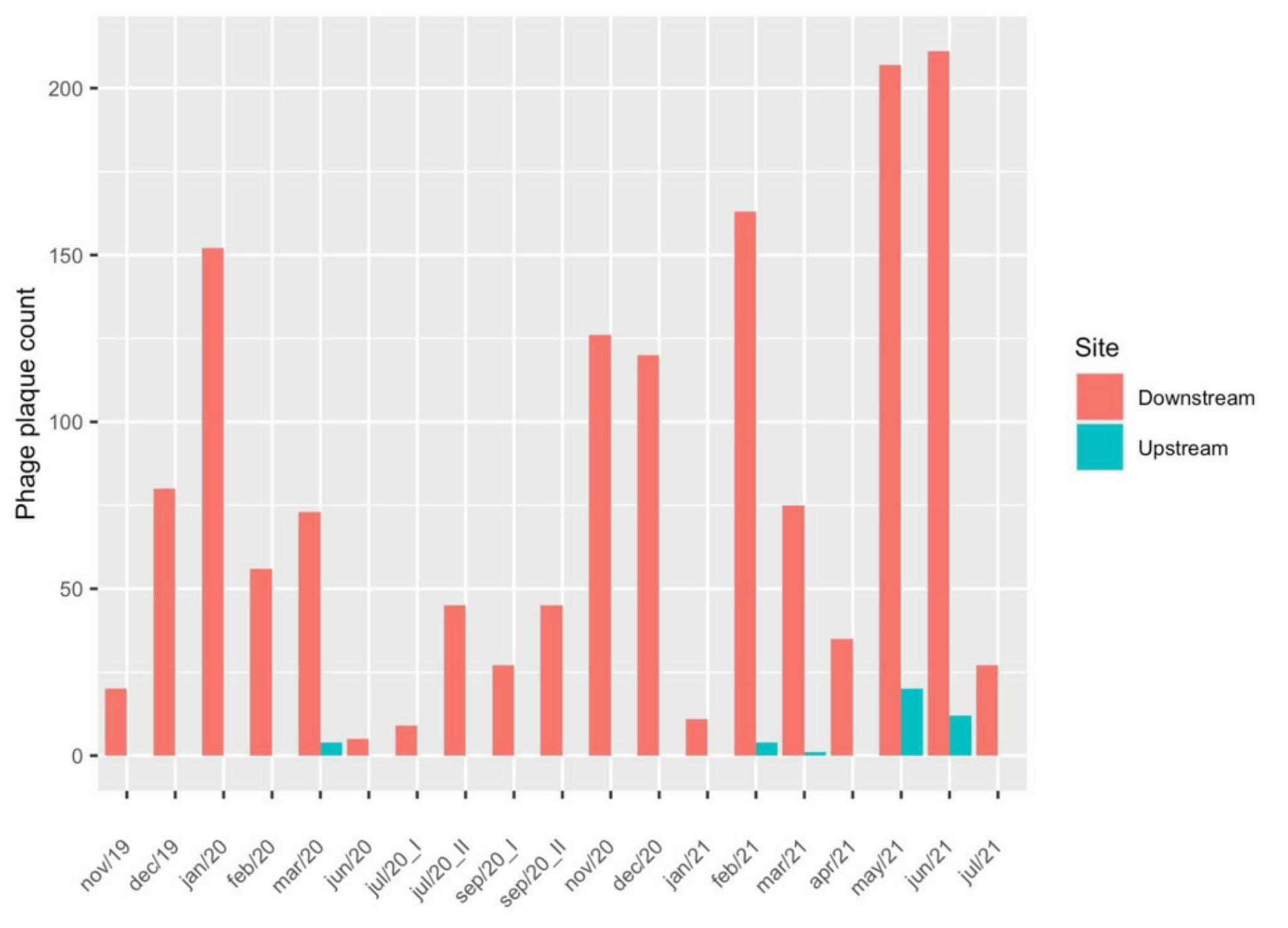

Supplement: S1 Fig — (TIF) [file pntd.0011912.s004.tif]

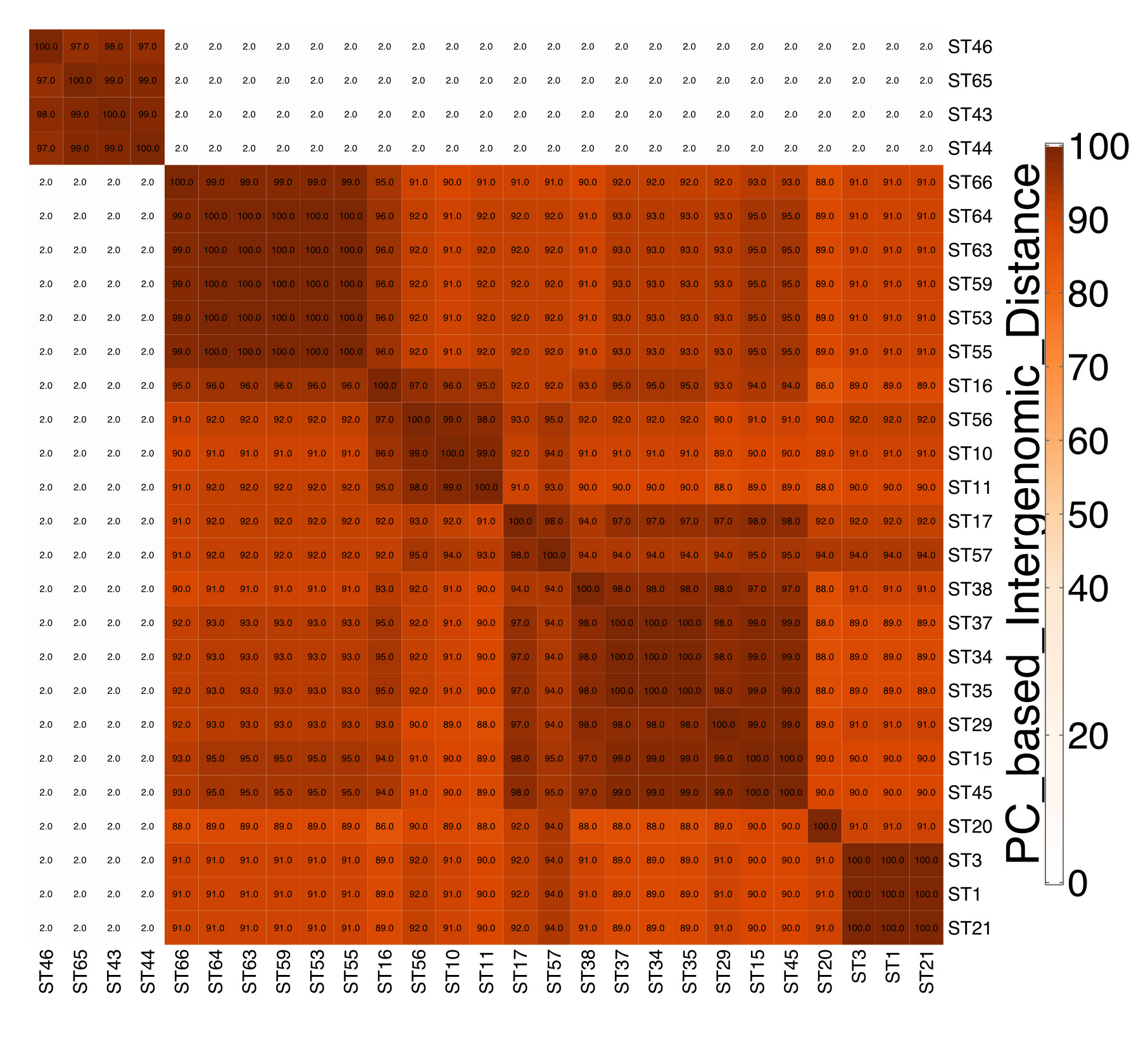

Supplement: S2 Fig — The numbers and colors indicate similarity between the phage genomes from none or lower (red) to high (dark red). (TIF) [file pntd.0011912.s005.tif]

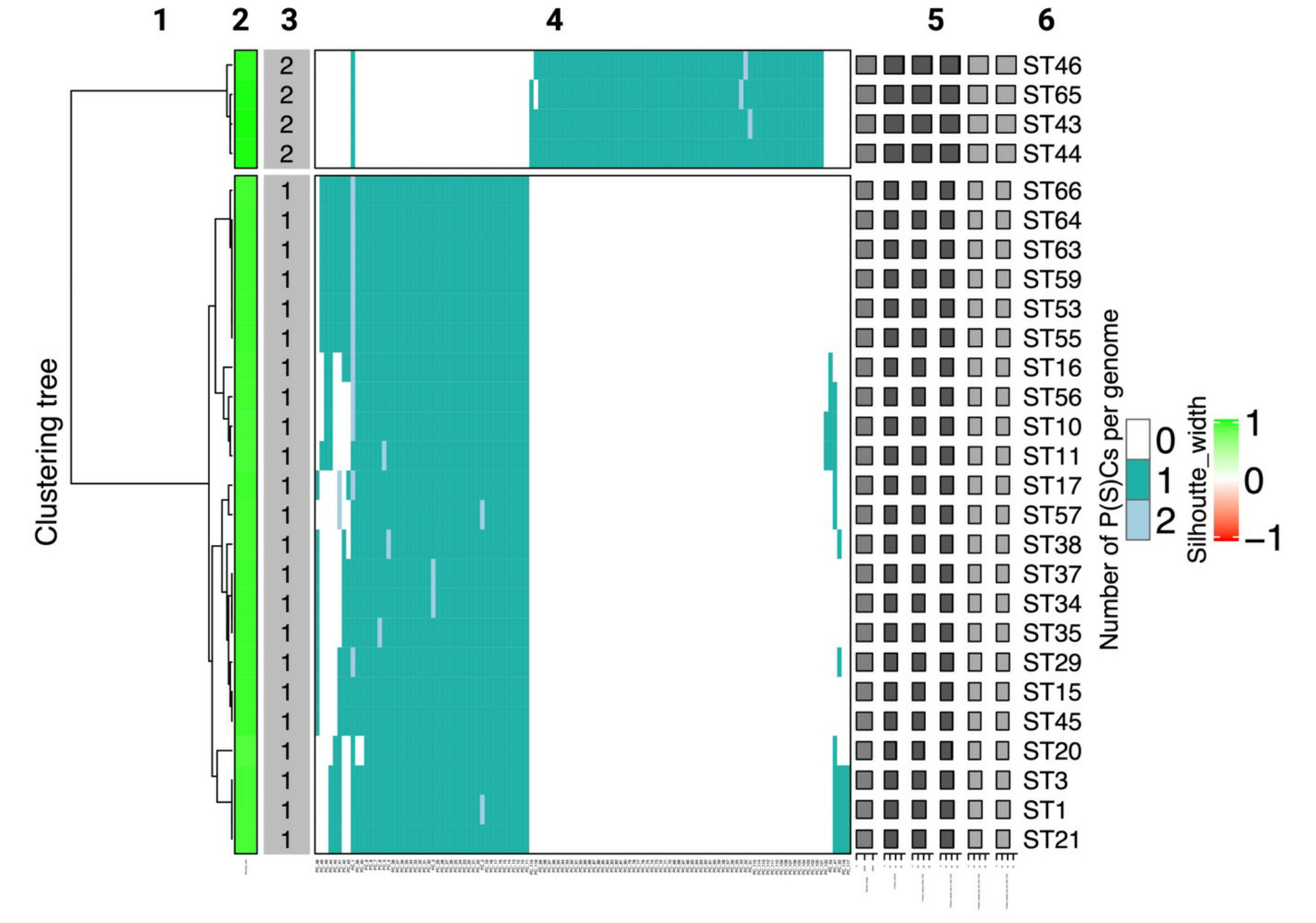

Supplement: S3 Fig — The genome clustering was performed based on PCs. The resulting tree was split into VGCs using a 0.9 intergenomic distance threshold. The visual components are described further. 1. Hierarchical tree calculated using PC-based intergenomic distances. 2. Silhouette width, color-coded in a range from −1 (red) to 1 (green). 3. VGC ID. 4. Heatmap representation of the PC distribution in the viral genomes. Rows are represented by individual viral genomes. Columns are represented by individual PCs. The ID of each PC can be read at the bottom of the heatmap. Colors encode the number of each PC per genome, with white signifying the PC absence, and the other colors signifying various degrees of replication. 5. Viral genome-specific statistics: genome length, the proportion of PC shared (dark grey) with any other genomes in the dataset, reported to the total PCs in the genome (light grey bar), the proportion of PC shared in its own VGC, the proportion of PCs shared only in its own VGC, the proportion of PCs shared also outside its own VGC, and the proportion of PC shared only outside own VGC. 6. S. Typhi phage name. (TIF) [file pntd.0011912.s006.tif]

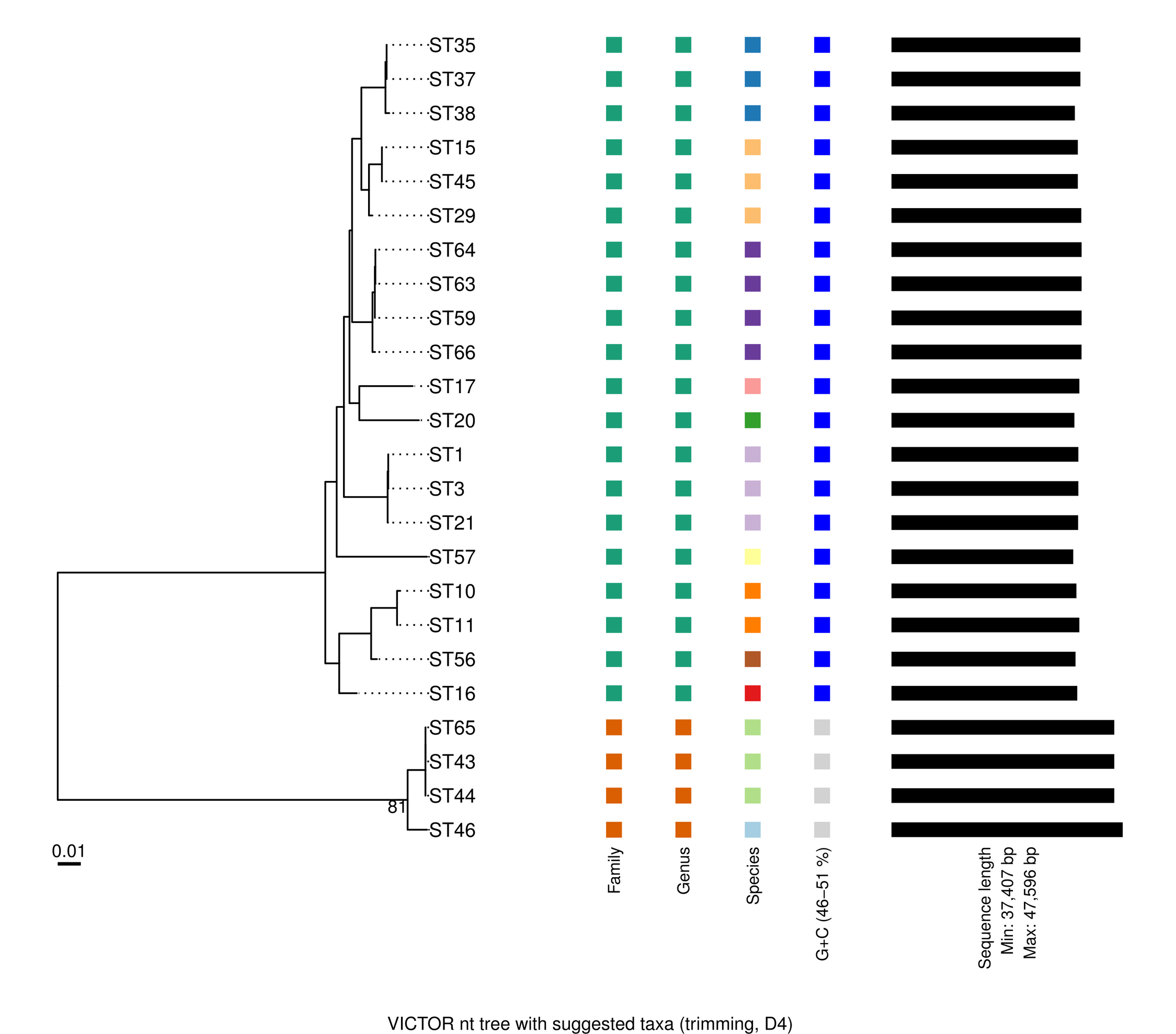

Supplement: S4 Fig — All pairwise comparisons of the nucleotide sequences were conducted using the Genome-BLAST Distance Phylogeny (GBDP) method under settings recommended for prokaryotic viruses using VICTOR software. The resulting intergenomic distances were used to infer a balanced minimum evolution tree with branch support via FASTME including SPR postprocessing. Branch support was inferred from 100 pseudo-bootstrap replicates each. Branches with bootstrap values below 50 were collapsed and the bootstrap values equal to or above 50 are shown on the remainder of the tree branches. (TIF) [file pntd.0011912.s007.tif]

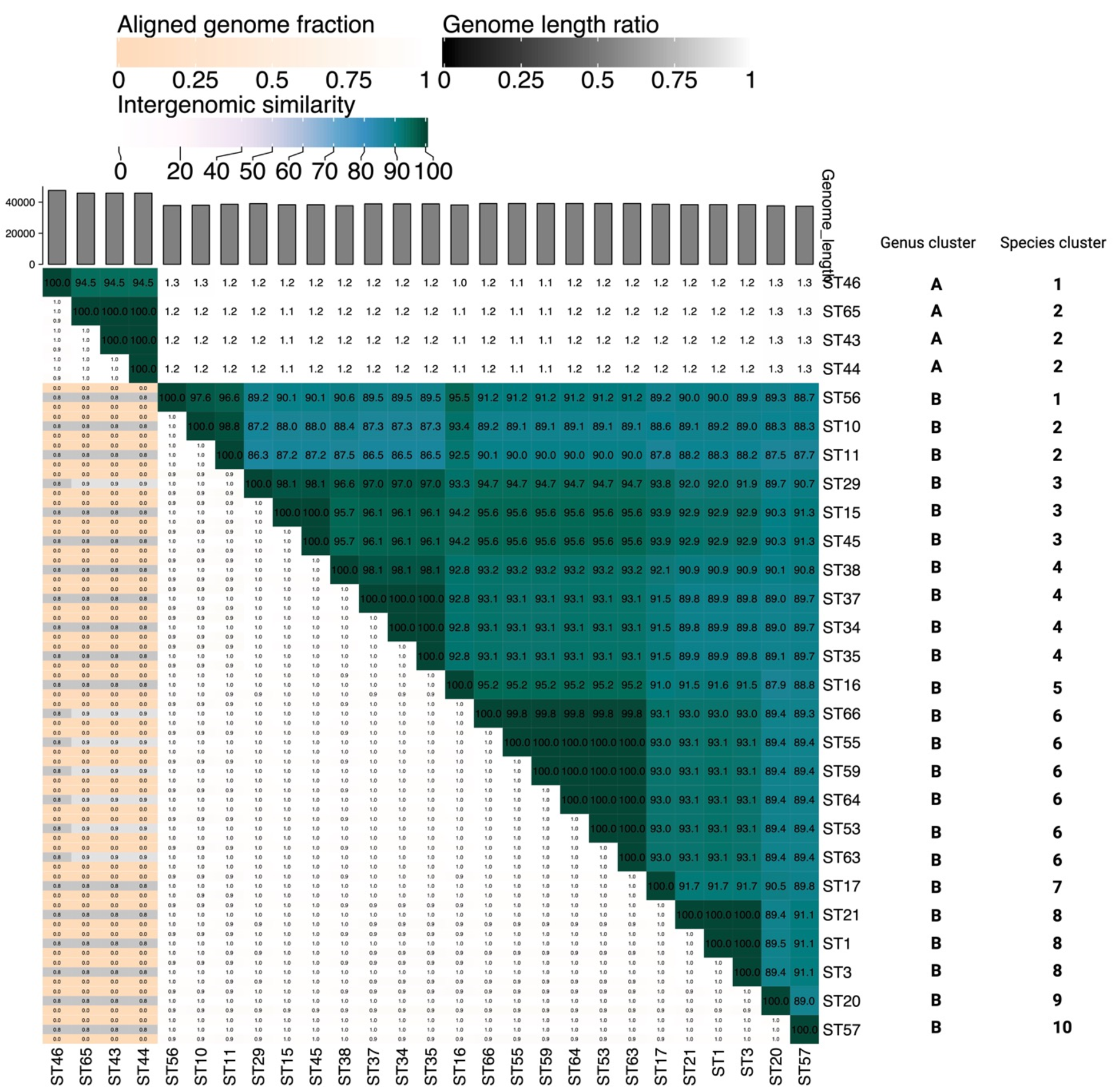

Supplement: S5 Fig — A heatmap of hierarchical clustering of the intergenomic similarity values was generated and given as percentage values (right half, blue-green heatmap). Each genome pair is represented by three values (left half), where the top and bottom represent the aligned genome fraction for the genome in the row and column, respectively. The middle value represents the genome length ratio for each genome pair. (TIF) [file pntd.0011912.s008.tif]
